# Development and external validation of a novel multihematoma fuzzy sign on computed tomography for predicting traumatic intraparenchymal hematoma expansion

**DOI:** 10.1038/s41598-021-81685-8

**Published:** 2021-01-21

**Authors:** Jiangtao Sheng, Jinhua Yang, Shirong Cai, Dongzhou Zhuang, Tian Li, Xiaoxuan Chen, Gefei Wang, Jianping Dai, Faxiu Ding, Lu Tian, Fengqing Zheng, Fei Tian, Mindong Huang, Kangsheng Li, Weiqiang Chen

**Affiliations:** 1 Department of Microbiology and Immunology and Key Immunopathology Laboratory of Guangdong Province, Shantou University Medical College, 22 Xinling Road, Shantou, Guangdong China; 2 Department of Neurosurgery, First Affiliated Hospital of Shantou University Medical College, 57 Changping Road, Shantou, Guangdong China; 3 Department of Neurosurgery, Second Affiliated Hospital of Shantou University Medical College, Shantou, Guangdong China; 4 Department of Neurosurgery, Affiliated Jieyang Hospital of Sun Yat-Sen University, Jieyang, Guangdong China

**Keywords:** Neurological manifestations, Trauma, Brain injuries, Risk factors

## Abstract

Acute traumatic intraparenchymal hematoma (tICH) expansion is a devastating neurological complication that is associated with poor outcome after cerebral contusion. This study aimed to develop and validate a novel noncontrast computed tomography (CT) (NCCT) multihematoma fuzzy sign to predict acute tICH expansion. In this multicenter, prospective cohort study, multihematoma fuzzy signs on baseline CT were found in 212 (43.89%) of total 482 patients. Patients with the multihematoma fuzzy sign had a higher frequency of tICH expansion than those without (90.79% (138) vs. 46.71% (71)). The presence of multihematoma fuzzy sign was associated with increased risk for acute tICH expansion in entire cohort (odds ratio [OR]: 16.15; 95% confidence interval (CI) 8.85–29.47; P < 0.001) and in the cohort after propensity-score matching (OR: 9.37; 95% CI 4.52–19.43; P < 0.001). Receiver operating characteristic analysis indicated a better discriminative ability of the presence of multihematoma fuzzy sign for acute tICH expansion (AUC = 0.79; 95% CI 0.76–0.83), as was also observed in an external validation cohort (AUC = 0.76; 95% CI 0.67–0.84). The novel NCCT marker of multihematoma fuzzy sign could be easily identified on baseline CT and is an easy-to-use predictive tool for tICH expansion in the early stage of cerebral contusion.

## Introduction

A devastating neurological complication of traumatic brain injury (TBI) is acute traumatic intraparenchymal hematoma (tICH), which is mainly caused by cerebral contusion. Clinically significant tICH expansion, which occurs in approximately 38–63% of patients with tICH^1–4^, is a determining factor of poor outcome. In contrast to non-modifiable outcomes factors, such as baseline hematoma volume and location of hematoma, acute tICH expansion is a distinctly important modifiable target. However, our ability for the early identification of patients who are most likely to experience acute tICH expansion remains limited.

Contrast extravasation (CE) on computed tomography (CT) angiography (CTA), which was originally described in the setting of spontaneous ICH (sICH), is a promising imaging predictor of tICH expansion^5–7^. However, this marker can only be evaluated using CTA, which is not a routine examination for TBI patients in emergency nor is widely available in hospitals, especially in less developed areas. In addition, CTA increases the radiation exposure of patients^8,9^. These disadvantages may limit the wide clinical applications of CE.

Noncontrast CT (NCCT) is an inexpensive and widely available tool for tICH diagnosis worldwide. Developing novel NCCT markers of tICH expansion can provide a broadly applicable tool for the timely recognition of contusion patients with a high risk of acute tICH expansion. On the basis of the association between spontaneous hematoma heterogeneity and hematoma growth, several heterogeneous hematoma markers including blend sign, black hole sign, and hypodensities on NCCT have been developed to predict sICH expansion^10–14^. However, no studies have systematically investigated the predictive value of traumatic hematoma heterogeneity for tICH expansion. Here, we developed and validated a novel traumatic heterogeneous hematoma marker of multihematoma fuzzy sign on NCCT upon admission for predicting acute tICH expansion.

## Patients and methods

The study, including any relevant details was approved by the ethics committees of the First Affiliated Hospital of Shantou University Medical College, the Second Affiliated Hospital of Shantou University Medical College, and the Affiliated Jieyang Hospital of Sun Yat-sen University. All methods were performed in accordance with the relevant guidelines and regulations. Informed consent for study inclusion was obtained from all patients (or their surrogates) before they participated in this study.

### Study population

**Figure 1:**
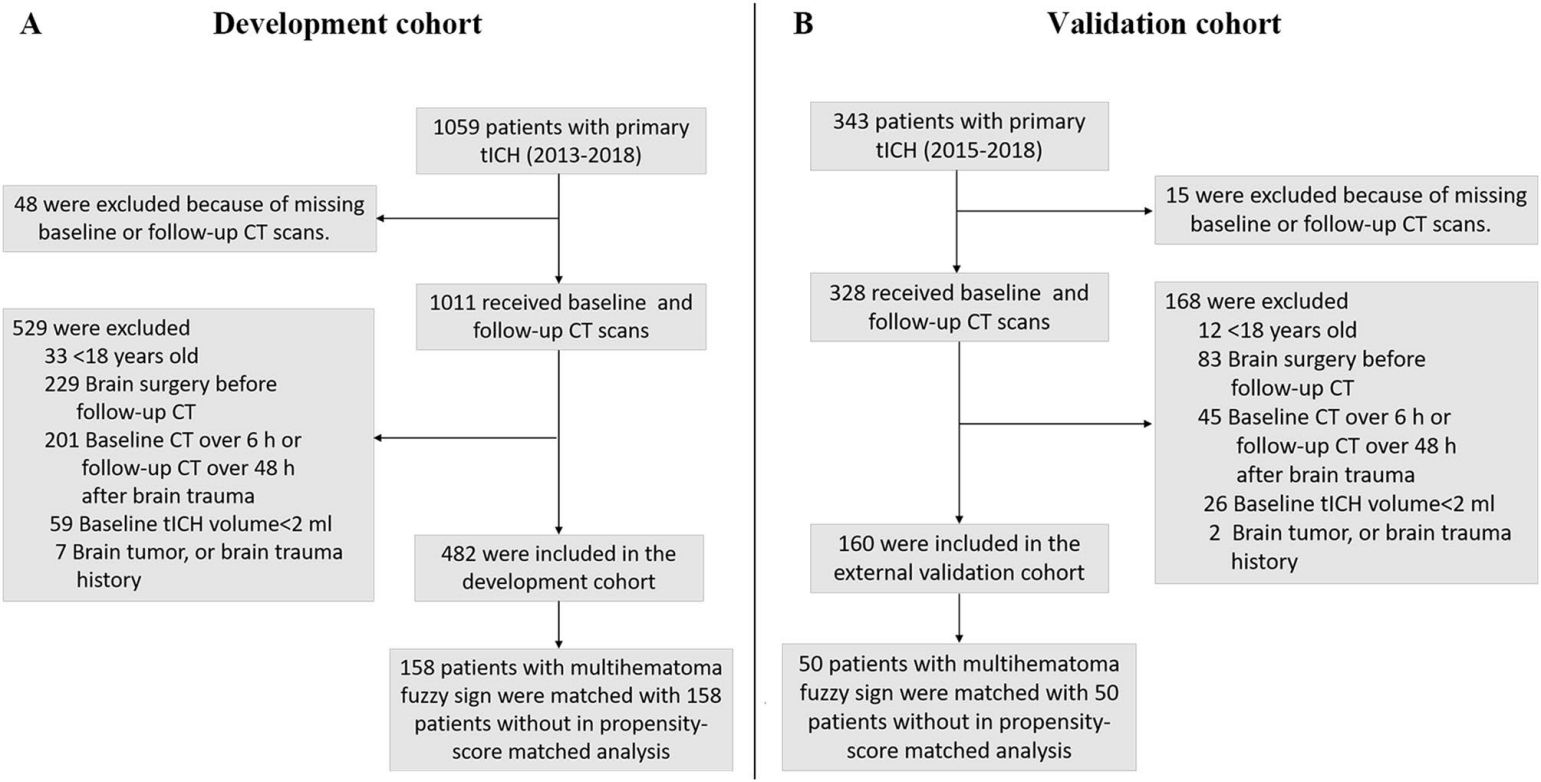
Flowchart of patient selection process including inclusion and exclusion criteria. Patients in the development cohort were selected from First Affiliated Hospitals of Shantou University Medical College and Second Affiliated Hospitals of Shantou University Medical College between May 2013 and June 2018 (**A**). Patients in the validation cohort were selected from Affiliated Jieyang Hospital of Sun Yat-sen University between March 2015 and June 2018 (**B**). *CT* noncontrast computed tomography.

Development cohort prospectively included patients aged > 18 years with primary hemorrhagic contusion and who underwent baseline and follow-up CT in First Affiliated Hospital of Shantou University Medical College, the Second Affiliated Hospital of Shantou University Medical College), between May 2013 and June 2018 (Fig. 1A). Patients in an external validation cohort were prospectively included from Affiliated Jieyang Hospital of Sun Yat-sen University, between March 2014 and June 2018 (Fig. 1B). All patients were treated in accordance with the standardized institutional protocol of each hospital during the recruitment period. Furthermore, tICH was confirmed on baseline CT showing intraparenchymal bleeding. Patients were excluded from the study if the baseline CT was over 6 h, or the follow-up CT was over 48 h after brain trauma. Patients were excluded if they had undergone surgical evacuation of hematoma before the follow-up CT. Patients were also excluded from the study if they had a brain tumor or a brain trauma history. In the development cohort, 5.57% of total patients with a baseline tICH volume of < 2 mL were also excluded from the study because of difficulties in accurately measuring hematoma volume^1^.

### Clinical data

We collected demographic and clinical data, as listed in Table 1. In addition, Patients were identified as having a coagulation disorder if activated partial thromboplastin time (aPTT) ≥ 36 s, international normalized ratio (INR) > 1.2, or platelet count < 120 × 10^9^ platelets/l at admission^15^.

**Table 1:** Baseline characteristics of patients with or without multihematoma fuzzy sign before and after propensity score (PS) matching.

Variables	Before PS match			After PS match		
	No multihematoma fuzzy sign (n = 270)	Multihematoma fuzzy sign (n = 212)	*P* value	No multihematoma fuzzy sign (n = 158)	Multihematoma fuzzy sign (n = 158)	*P* value
Male sex, no. (%)	204 (75.56%)	157 (74.06%)	0.706	115 (72.78%)	117 (74.05%)	0.799
Mean age (SD), y	50.99 (17.22)	50.60 (18.17)	0.808	51.32 (17.47)	50.89 (17.43)	0.827
Hypertension, no. (%)	29 (11.28%)	32 (15.69%)	0.166	18 (11.39%)	21 (13.29%)	0.608
Diabetes, no. (%)	15 (5.68%)	15 (7.25%)	0.490	10 (6.33%)	10 (6.33%)	1.000
Mean arterial pressure, median (IQR) (mmHg)	100.00 (90.75–109.83)	103.00 (94.00–113.66)	0.008	100.00 (91.00–110.00)	100.00 (91.75–110.00)	0.698
Coagulopathy, no. (%)	42 (16.03%)	35 (16.91%)	0.799	26 (16.46%)	27 (17.09%)	0.880
**Level on Glasgow Coma Scale score, no. (%)**			0.241			0.858
Mild (13–15 points)	142 (52.59%)	100 (47.17%)		81 (51.27%)	83 (52.53%)	
Moderate (9–12 points)	53 (19.63%)	55 (25.94%)		36 (22.78%)	32 (20.25%)	
Severe (3–8 points)	75 (27.78%)	57 (26.89%)		41 (25.95%)	43 (27.22%)	
**Location, no. (%)**			< 0.001			0.974
Frontal	109 (40.37%)	125 (58.96%)		86 (54.43%)	80 (50.63%)	
Temporal	127 (47.04%)	76 (35.85%)		61 (38.61%)	67 (42.41%)	
Parietal	9 (3.33%)	7 (3.30%)		7 ( 4.43%)	7 ( 4.43%)	
Occipital	12 (4.44%)	2 (0.94%)		2 ( 1.27%)	2 ( 1.27%)	
Basal ganglia, brainstem, or cerebellum	13 (4.81%)	2 (0.94%)		2 ( 1.27%)	2 ( 1.27%)	
Intraventricular hemorrhage, no. (%)	22 (8.15%)	18 (8.49%)	0.892	11 ( 6.96%)	11 ( 6.96%)	1.000
Subarachnoid hemorrhage, no. (%)	213 (78.89%)	189 (89.15%)	0.003	137 (86.71%)	136 (86.08%)	0.870
Subdural hemorrhage, no. (%)	189 (70.00%)	180 (84.91%)	< 0.001	127 (80.38%)	127 (80.38%)	1.000
Time to baseline CT (IQR) (h)	2.50 (1.50–4.00)	2.17 (1.50–4.00)	0.148	2.37 (1.50–4.27)	2.60 (1.67–4.24)	0.672
Time from baseline CT to follow-up CT (IQR) (h)	16.66 (9.87–24.00)	16.25 (8.59–24.00)	0.967	15.59 (7.08–24.00)	17.00 (8.10–24.00)	0.490
Baseline tICH volume, mean (SD) (ml)	7.70 (8.29)	10.96 (11.10)	< 0.001	8.29 (8.99)	8.96 (7.42)	0.470

### Imaging data analysis

**Figure 2:**
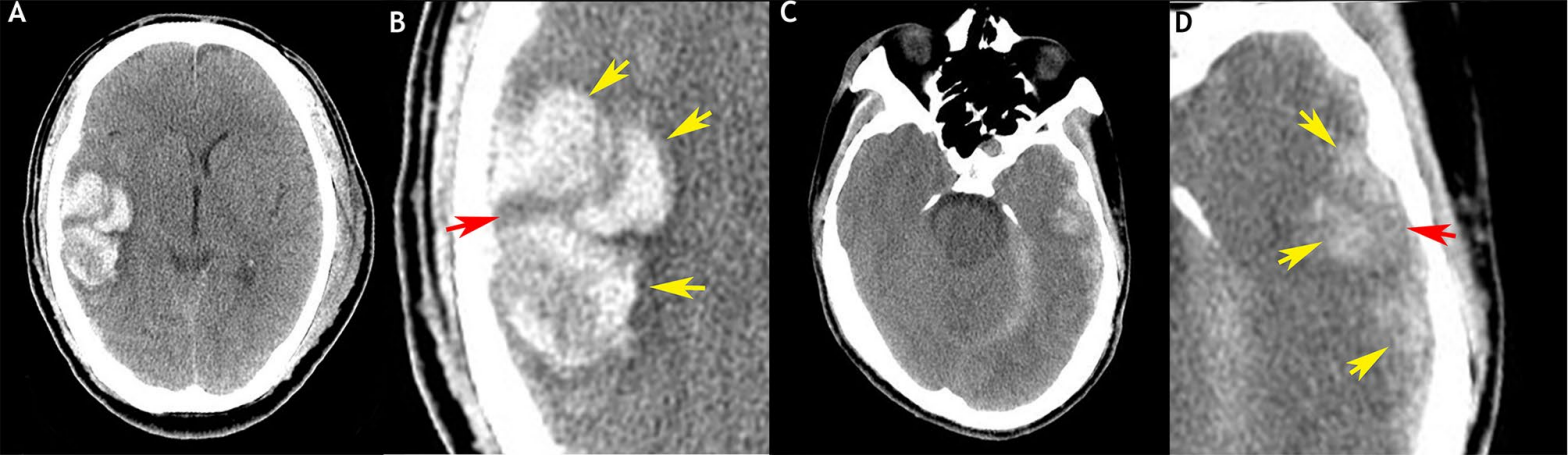
Illustration of multihematoma fuzzy signs on baseline CT, showing two representative multihematoma fuzzy signs in the contusion region (**A**, **C**) and their respective local zones (**B**, **D**). Axial sections of CT reveal three relative hyperdense hematomas adjacent to each other (yellow arrows) in the contusion regions and the presence of a relative hypodense fuzzy signal (red arrows) on the area between the hematomas.

In this study, multihematoma fuzzy sign was defined as follows. (1) For multiple hyperdense hematomas (≥ 3 hematomas) adjacent to each other in the contusion region, the maximum distance of separation between hematomas before they are considered unrelated is the largest diameter of the largest hematoma. (2) For a relative hypodense fuzzy signal on the area between the hyperdense hematomas, the fuzzy sign can be regarded as the burring of hyperdense hematomas (from the perspective of image interpretation, the fuzzy possibly indicates fresh liquid blood); (3) the difference between hyperdense hematomas and the hypodense fuzzy area is ≥ 20 HU. Multiple adjacent hematomas in the contusion regions must meet all three criteria mentioned above to be defined as a multihematoma fuzzy sign (Fig. 2A–D). Notably, multiple hyperdense hematomas separated by relatively hypodense gray/white matter must not be considered a multihematoma fuzzy sign.

Axial NCCT images were obtained at each participant’s institution by using standardized local protocols. Baseline CT images with 5 mm slice thickness were reviewed by two readers (D.Z. and S.C.) who were blinded to other clinical data. Discrepancies regarding the occurrence of multihematoma fuzzy sign were reviewed by a senior neuroradiologist (J.Y.) blinded to previous judgment and who provided a final interpretation. Baseline and follow-up hematoma volumes were calculated from the CT images via semiautomated computer-assisted volumetric analysis (General Electric Company, Waukesha, USA)^16^. First, the region of interest was selected by manual selection and automatically separated from the environment via a software on the basis of a fixed threshold in Hounsfield units (HU). The isolated regions were visually inspected and manually adjusted to ensure that the hemorrhage was visible in all three projections. Adjacent voxels were automatically summarized, thereby providing the hematoma volume, by using a threshold value for distinguishing hematomas from the surrounding brain tissue. CT images were assessed using a fixed window of 110 and 50 HU. When multiple ICHs were present in the contusion region, the total volume was calculated. Acute tICH expansion was defined as a relative growth of ≥ 30% or absolute hematoma growth of ≥ 5 mL from the initial CT as previously described^17,18^.

### Statistical analysis

Continuous variables are presented as means (standard deviations) or medians (interquartile ranges). Categorical variables are presented as counts (percentages). Independent associations between multihematoma fuzzy sign and other risk factors and tICH expansion were analyzed using multivariate logistic regression.

In addition, we constructed a propensity score for adjustment and matching. In the entire development cohort, propensity score was estimated with the use of a nonparsimonious multivariable logistic-regression model^19–21^, with the multihematoma fuzzy sign as the dependent variable and all baseline characteristics outlined in Table 1 as covariates. Propensity score matching was performed with a 1:1 matching protocol without replacement (greedy-matching algorithm) with a caliper width equal to 5% for propensity scores. Standardized differences were estimated for all the baseline covariates before and after matching to assess prematch imbalance and postmatch balance^21^. Standardized differences of less than 10.00% for a given covariate indicate a relatively small imbalance^22^.

In the matched cohort, paired comparisons were performed with the use of McNemar’s test for binary variables and paired Student’s t-test or paired-sample test for continuous variables. The comparative risks of tICH expansion were further adjusted in the matched cohort with the use of a Cox proportional-hazards regression model that was stratified on the matched pair to preserve the benefit of matching^23–25^. To further assess the robustness of our results, we performed sensitivity analyses by testing the association between the multihematoma fuzzy sign and acute tICH expansion only in patients with multiple hematomas (≥ 3 hematomas). In the matched cohort, interaction tests and subgroup analyses were used to further assess potential heterogeneity of the multihematoma fuzzy sign on tICH expansion^26^. Predefined subgroups included male versus female sex, age younger than 65 years versus no less than 65 years, mild GCS scores of 13–15 versus moderate and severe GCS scores of 3–12, less than 10 ml versus no less than 10 ml baseline tICH volume, 0–3 versus 3–6 h time from onset of brain trauma to baseline CT scan, and normal coagulation function versus coagulopathy after brain contusion.

Finally, receiver operating characteristic (ROC) curve analysis was performed to examine the discriminative ability of the multihematoma fuzzy sign and other risk factors for tICH expansion in the pre-matched cohort. All tests of significance were two-tailed, and *P* < 0.05 was considered statistically significant. All analyses were performed using SPSS version 22 (SPSS Inc., Chicago, Illinois, USA). This report was prepared following the Strengthening the Reporting of Observational Studies in Epidemiology guidelines^27^.

## Results

### Patient characteristics

**Figure 3:**
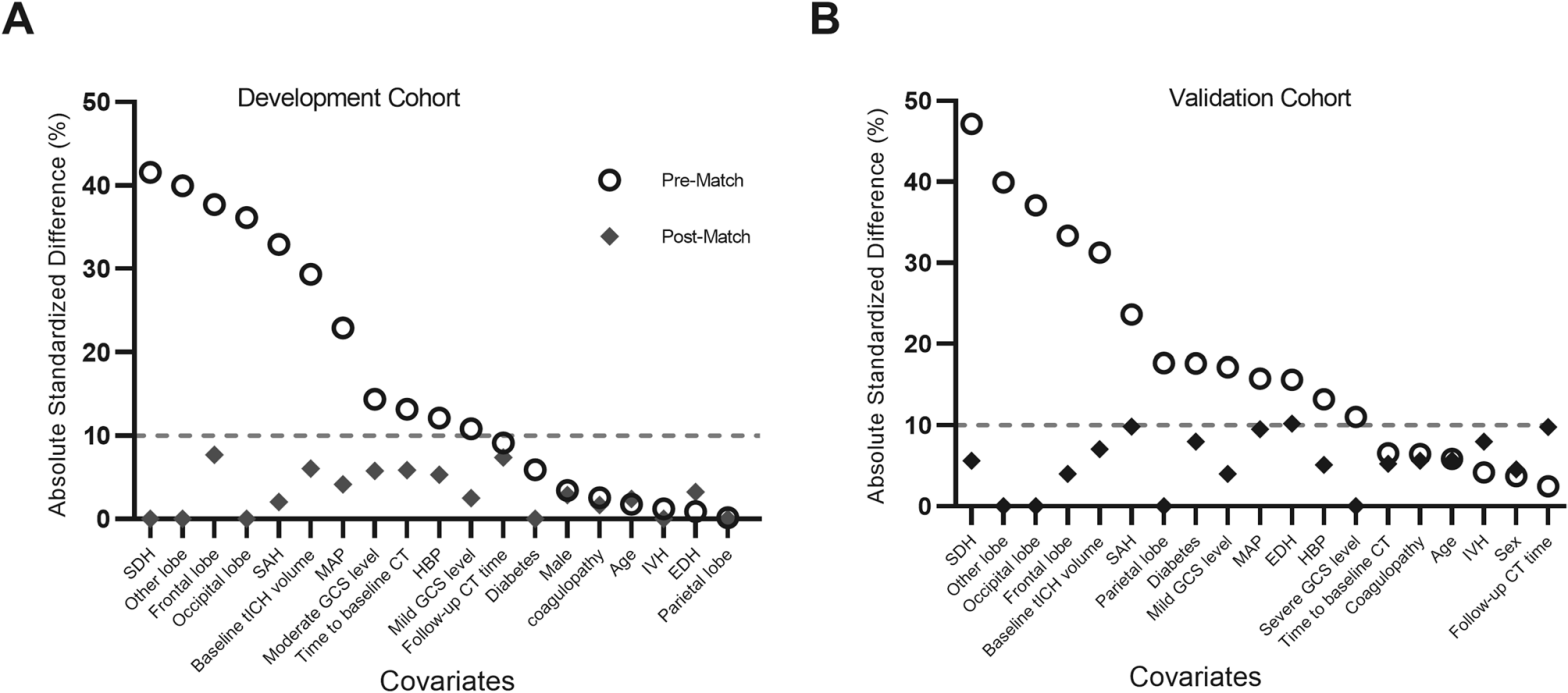
Absolute standardized differences before and after propensity score matching comparing variables values for patients with and without the multihematoma fuzzy sign in the development (**A**) and external validation cohorts (**B**).

Based on eligibility criteria, 482 out of 1059 patients with tICH were included in the development cohort (Fig. 1A). Among these patients, 212 (43.89%) demonstrated multihematoma fuzzy sign on the baseline CT, and 307 (63.69%) patients with tICH had significant acute tICH expansion. Before propensity score matching, patients with multihematoma fuzzy signs were more likely to have a large baseline hematoma volume, a higher mean arterial pressure and high values for frequency of frontal lobe tICH, subarachnoid hemorrhage, subdural hemorrhage (Table 1). With the use of propensity score matching, 158 patients with multihematoma fuzzy sign on the baseline CT with were matched with 158 patients without. After matching, the two groups were similar with regard to all the baseline variables (Table 1). The standardized differences were less than 10.0% for all variables, indicating substantial improvement in variable balance between the two groups (Fig. 3A).

### Main analyses

After matching, 209 (68.75%) patients with tICH had are acute tICH expansion. Compared with 71 (46.71%) tICH expansion patients without multihematoma fuzzy sign group, 138 (90.79%) of those in the multihematoma fuzzy sign group exhibited a tICH expansion. As summarized in Table 2, the presence of multihematoma fuzzy sign was associated with the high risk of acute tICH expansion (odds ratio (OR): 16.15; 95% CI 8.85–29.47; P < 0.001; Table 2) in our crude analysis. The association remained significant after adjusting for individual confounders (OR: 15.32; 95% CI 8.89–26.37; P < 0.001), adjustment by propensity score (OR: 12.42; 95% CI 7.14–21.63; P < 0.001), and propensity score matching (OR: 9.37; 95% CI 4.52–19.43; P < 0.001).

**Table 2:** Odds ratios associated with tICH expansion, and the presence of multihematoma fuzzy sign in the development cohort and validation cohort.

Adjusted model	Development cohort		Validation cohort	
	Odds ratio (95% CI)	*P* value	Odds ratio (95% CI)	*P* value
Adjusted by sex and age	16.15 (8.85, 29.47)	< 0.001	12.66 (3.63–44.20)	< 0.001
Adjusted by individual confounders	15.32 (8.89, 26.37)	< 0.001	10.95 (4.16, 28.83)	< 0.001
Adjusted by propensity score	12.42 (7.14, 21.63)	< 0.001	9.58 (3.80, 24.18)	< 0.001
Propensity score matched	9.37 (4.52, 19.43)	< 0.001	7.88 (3.06, 20.25)	< 0.001

### Sensitivity and subgroup analyses

We repeated the primary analysis in patients with multiple hematoma (≥ 3 hematomas). The presence of multihematoma fuzzy sign became weak but significant with tICH expansion (OR: 12.79; 95% CI 6.29–25.98; P < 0.001; Table 3) when adjusted by individual confounders (OR: 11.55; 95% CI 5.50–24.28; P < 0.001), by propensity score (OR: 10.51; 95% CI 4.85–22.76; P < 0.001), and by propensity score matching (OR: 7.00; 95% CI 1.59–30.83; P = 0.008).

**Table 3:** Odds ratios associated with tICH expansion, and the presence of multihematoma fuzzy sign in patients with multiple hematomas (≥ 3 hematomas).

Adjusted model	Development cohort		Validation cohort	
	Odds ratio (95% CI)	*P* value	Odds ratio (95% CI)	*P* value
Adjusted by sex and age	12.79 (6.29, 25.98)	< 0.001	7.95 (1.78, 35.60)	< 0.001
Adjusted by individual confounders	11.55 (5.50, 24.28)	< 0.001	6.75 (2.69, 49.13)	< 0.001
Adjusted by propensity score	10.51 (4.85, 22.76)	< 0.001	6.20 (2.49, 19.18)	0.008
Propensity score matched	7.00 (1.59, 30.80)	< 0.001	5.37 (2.33, 15.36)	0.023

**Figure 4:**
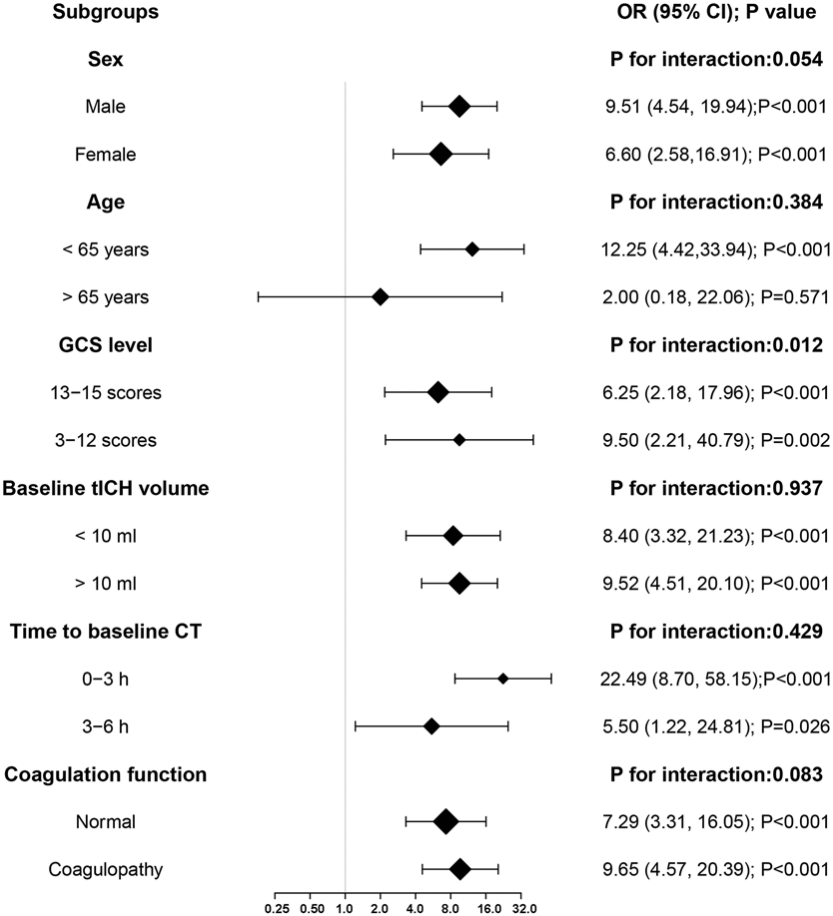
OR (95% CI) for acute tICH expansion in subgroups of cerebral contusion patients and interaction test of stratification factors and the multihematoma fuzzy sign in the matched cohort.

In the matched cohort, further subgroups analysis indicated that the association of the presence of multihematoma fuzzy sign with tICH expansion occurred across a wide spectrum of patients with brain contusion (Fig. 4). No significant interaction was observed between the presence of multihematoma fuzzy sign and any of the covariates, except for the GCS level (P for interaction = 0.012). The risk increase for tICH expansion was more substantial in moderate and severe brain contusion patients (OR: 9.50; 95% CI 2.21–40.79; P = 0.002) than in mild brain contusion patients (OR: 6.25; 95% CI 2.18–17.96; P < 0.001).

### ROC analysis

**Figure 5:**
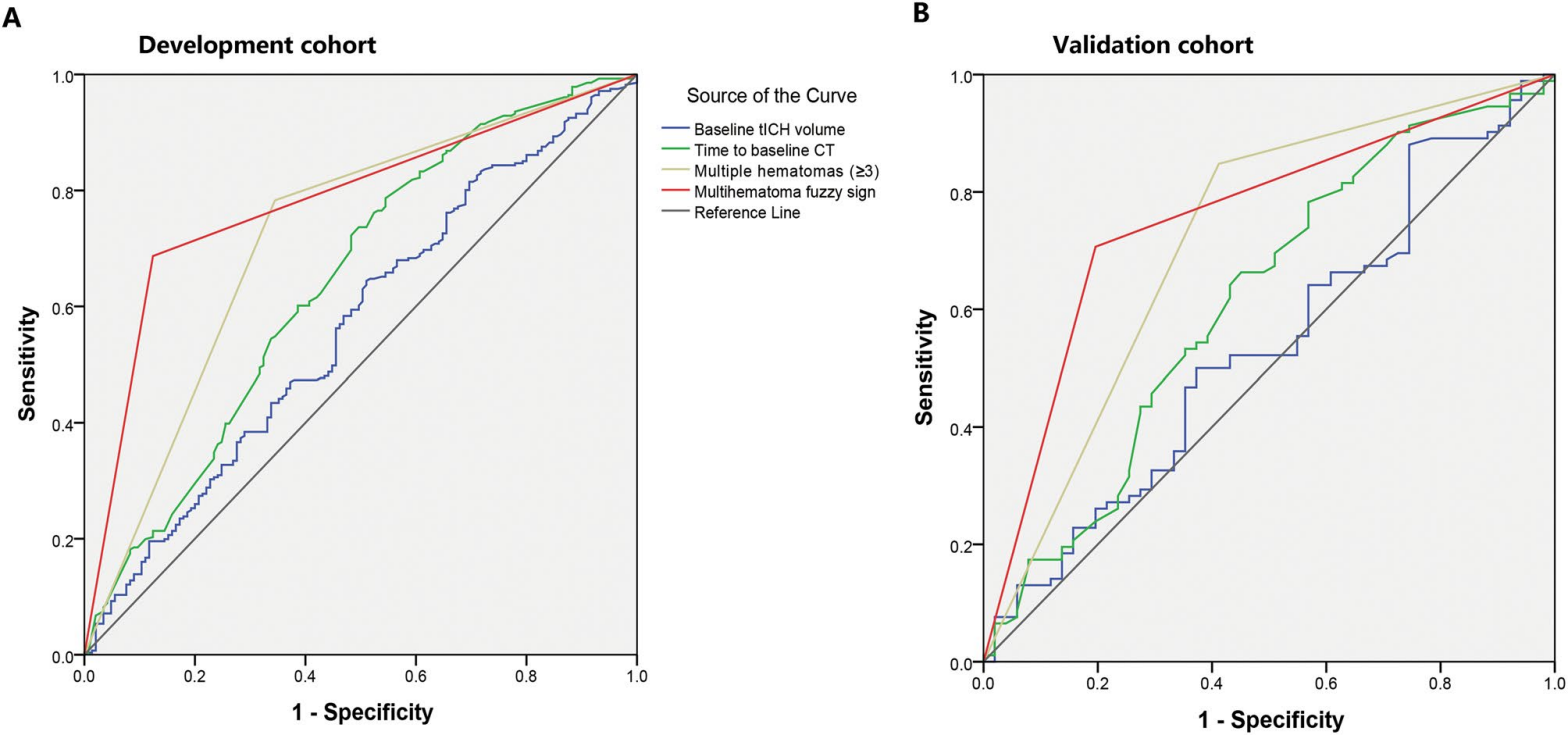
ROC curves for baseline tICH volume, time to baseline CT, the presence of multiple hematomas, and the presence of multihematoma fuzzy sign in predicting acute tICH expansion in the development (**A**) and validation cohorts (**B**).

In the entire cohort, we further compared the predictive value of the presence of multihematoma fuzzy sign with other risk factors, including multiple hematomas, time to baseline CT, and baseline hematoma volume for acute tICH expansion, by using ROC curve analysis (Fig. 5A). Area under the curve (AUC) for the presence of multihematoma fuzzy sign (AUC: 0.79; 95% CI 0.76–0.83) was larger than that for simple multiple hematomas (AUC: 0.71; 95% CI 0.67–0.77), time to baseline CT (AUC: 0.64; 95% CI 0.59–0.70), and baseline tICH volume (AUC: 0.57; 95% CI 0.51–0.62) for predicting acute tICH expansion.

### External validation

After matching of the external validation cohort, 50 patients with multihematoma fuzzy sign on the baseline CT were matched with 50 patients without (Fig. 1B). Acute tICH expansion occurred in 102 of the 160 (63.75%) patients with tICH. Before propensity score matching, patients with multihematoma fuzzy signs were likely to have a large baseline hematoma volume and a high frequency of frontal lobe tICH. After matching, 50 patients with multihematoma fuzzy sign on the baseline CT were matched with 50 patients without. The two groups were similar with regard to all of the baseline variables (Supplementary Table 1 and Fig. 3B). Compared with 22 (44.00%) tICH expansion patients of the no-multihematoma-fuzzy-sign group, 44 (88.00%) of those in the multihematoma-fuzzy-sign group presented tICH expansion. Consistently, the presence of multihematoma fuzzy sign was associated with tICH expansion in the matched cohort (OR: 7.88; 95% CI 3.06–20.25; P < 0.001; Table 2) and in patients with multiple hematomas (OR: 5.37; 95% CI 2.33–15.36; P = 0.023; Table 3). In ROC analysis, the presence of multihematoma fuzzy sign retained the highest AUC (AUC: 0.76; 95% CI 0.67–0.84) compared with multiple hematomas (AUC: 0.71; 95% CI 0.63–0.81), time to baseline CT (AUC: 0.62; 95% CI: 0.52–0.71), and baseline tICH volume (AUC: 0.54; 95% CI 0.44–0.64) for acute tICH expansion (Fig. 5B).

## Discussion

In this prospective observational study, we presented and validated a novel and easy-to-use imaging marker for predicting tICH growth. We demonstrated that the presence of multihematoma fuzzy sign on NCCT significantly increased the risk of acute tICH expansion across a wide spectrum of brain contusion patients. Compared with other risk factors, including simple multiple hematomas, time to baseline CT, and baseline CT volume, multihematoma fuzzy sign showed an optimal discriminative ability for acute tICH expansion.

In fact, multihematoma fuzzy sign refers to the appearance of hematoma heterogeneity and comprises CT with mixed densities of blood in the contusion region. Several studies have reported the association of hematoma heterogeneity with acute hematoma growth. In the 1980s, hematoma heterogeneity was observed in patients with traumatic epidural hemorrhage^28,29^, and the mixed density of hematomas has been reported to correlate with active bleeding, which has been validated during the surgical exploration of patients with epidural hemorrhage^30,31^. Of note, similar to the multihematoma fuzzy sign, fukamachi A, et al. described a characteristic salt and pepper or flecked patthern of mixed area of hypodensity and hyperdensity, which usually indicates delayed intracranial hematoma after head injury^32^. Recently, several hematoma heterogeneity markers including “hypodensities”, “blend sign” and “black hole sign” were developed to predict spontaneous intracerebral hematoma expansion^10–13^. Consistently, our study developed and validated a novel hematoma heterogeneity marker specific for traumatic cerebral parenchymal hematoma expansion. Moreover, subgroups analysis indicated that the association occurred across a wide spectrum of brain contusion patients. Notably, the marker in patients with moderate and severe cerebral contusion demonstrated a significantly stronger association with acute tICH expansion than in mild cerebral contusion patients (P for interaction = 0.012). This finding suggests that strengthening of medical management that prevents hematoma expansion should be given to patients with moderate and severe cerebral contusion and multihematoma fuzzy sign. The group with the fuzzy sign to have more frontal hematomas, but interaction test indicates that the association between hematoma location and the fuzzy sign was no significant (P = 0.104). Even so, we cannot rule out the role of hematoma location as a potential effect modifier of the multihematoma fuzzy sign. In this study, our data was likely underpowered to detect a significant interaction between the fuzzy sign and hematoma location.

From the perspective of image interpretation, the multihematoma fuzzy sign may reflect the coexistence of different stages of bleeding^33,34^. The density of blood on CT is dependent on the time course of bleeding. A crucial factor for hematoma density is hemoglobin, and other components of hematoma may show negligible effect^35^. Fresh liquid blood is hypodense on CT. Once clot retraction occurs, the serum is sequestered from the hematoma, making the hematoma appear hyperdense on CT. The presence of multihematoma fuzzy sign may suggest the coexistence of blood clot (hyperdense hematomas) and fresh liquid blood (hypodense fuzzy area), resulting in a heterogeneous appearance of the contusion region on CT. The presence of fresh liquid blood may explain the predictive potential of multihematoma fuzzy sign for acute tICH growth.

In the perspective of imaging marker for tICH expansion, three small cohort studies reported that the CE on CTA originally described in the context of sICH is a potential predictor for traumatic hematoma growth^5–7^. Marcos et al. observed that the AUC of CE was 0.80 (95% CI 0.7–0.9) for predicting tICH expansion^5^. The AUC of multihematoma fuzzy sign for predicting tICH expansion in our study was 0.79 (95% CI 0.76–0.83), matching the AUC of CE reported by Marcos et al. Of note, CTA is not a routine test nor actively used in many hospitals in the acute care scenario and increased radiation exposure^8,9^. Compared with the CTA marker of CE, the NCCT marker of multihematoma fuzzy sign may be more widely applicable in clinical practice. In addition, the discriminative ability of multihematoma fuzzy sign for tICH expansion is better than that of other potential predictors (multiple hematomas, baseline CT time, and baseline tICH volume). Thus, compared with other imaging markers or clinical indicators, the multihematoma fuzzy for predicting tICH expansion achieves a good balance between accuracy and convenience.

The presence of multiple hematomas is associated with traumatic hematoma expansion^36,37^. Simple multiple hematomas may cause confusion regarding the association between multihematoma fuzzy sign and tICH expansion. To verify the robustness of the association between the presence of multihematoma fuzzy sign and tICH expansion, we further analyzed the main results in cerebral contusion patients with multiple hematomas (≥ 3 hematomas). Whether in development-matched cohort or in validation-matched cohort, the presence of multihematoma fuzzy sign became weak but significant with tICH expansion. In ROC analysis, multihematoma fuzzy sign also exhibited a better discriminative ability on tICH expansion than simple multiple hematomas on tICH expansion. Therefore, the confounding effects of simple multiple hematoma, if any, cannot fully explain the association observed in this study. The association between the presence of between multihematoma fuzzy sign and tICH expansion is more substantial than that between simple occurrence of multiple hyperdense hematomas and tICH expansion.

Some of limitations should be considered in this study. To reduce the confounding bias, propensity score adjustment and propensity score matching were used to balance the difference in baseline characteristics between the patients with multihematoma fuzzy sign and those without. Even so, the bias from those unmeasured variables cannot be balanced. In this study, lifestyle factors, such as body mass index, smoking and alcohol drinking were lacked. We also failed to capture certain variables that may significantly affect hematoma growth, such as the use of anticoagulants before brain trauma. We adjusted this factor by including coagulation function upon admission. While, subgroups analysis found no significant effect of coagulopathy on the association between the multihematoma fuzzy sign and tICH expansion. One possible explanation for our finding is that our data analysis was underpowered to detect a significant association between coagulopathy and tICH growth. In addition, despite the evidence of a large effect, the confidence intervals are quite wide. Therefore, there is uncertainty about the effect of multihematoma fuzzy sign on acute tICH expansion.

Another limitation of our study was that we did not compare the capability to predict tICH expansion between the multihematoma fuzzy sign with existing imaging markers. First, the NCCT imaging markers for spontaneous hemorrhage may be unsuitable for predicting tICH expansion because of the difference between spontaneous and traumatic intraparenchymal hemorrhage on hemorrhage mechanism and hematoma morphology. These existing NCCT markers are uncommon on the CT scans of patients with cerebral contusion. Second, CTA imaging is not a routine examination for TBI patients in hospitals, and the vast majority of patients lack data of CE on CTA scan in our cohort. Additional cohort studies are needed to compare the capability to predict tICH expansion between the multihematoma fuzzy sign and CE.

Finally, the evaluation of the multihematoma fuzzy sign was not completely objective. Given individual differences on CT among the study participants, finding an optimal HU range for each region was difficult. At least 20 HU difference between the hematomas and the fuzzy area contributed in identifying the presence of hematoma heterogeneity. Other thresholds have also been tested, and similar predictive values of the multihematoma fuzzy sign were acquired. Meanwhile, a further analysis based on radiomics or deep mechanical learning will help eliminate the subjectivity of multihematoma fuzzy sign.

In summary, we developed and externally validated a novel NCCT marker for acute tICH expansion. The presence of multihematoma fuzzy sign significantly increased the risk of acute tICH expansion crossing a wide spectrum of brain contusion patients. Multihematoma fuzzy sign provides an easy-to-use CT marker for acute tICH expansion in patients with cerebral contusion.

## Supplementary Information

Supplementary Table 1.

## Data Availability

All data generated or analysed during this study are included in this published article (and its “Supplementary Information” files).
