# Supplementary material for: Development and external validation of a novel multihematoma fuzzy sign on computed tomography for predicting traumatic intraparenchymal hematoma expansion
Source: Sci Rep. 2021 Jan 21;11:2042. doi: 10.1038/s41598-021-81685-8 (PMC7819987; doi:10.1038/s41598-021-81685-8)
Supplement: Supplementary file 1 — Supplementary Table 1. [file 41598_2021_81685_MOESM1_ESM.docx]

**Development and External Validation of A Novel Multihematoma Fuzzy Sign on Computed Tomography for Predicting Traumatic Intraparenchymal Hematoma Expansion**

Jiangtao Sheng^1^, Jinhua Yang^2^, Shirong Cai^2^, Dongzhou Zhuang^2^, Tian Li ^1^, Xiaoxuan Chen^1^, Gefei Wang^1^, Jianping^1^, Faxiu Ding^2^, Lu Tian^1^, Fengqing Zheng^1^, Fei Tian^3^, Mindong Huang^4^, Kangsheng Li^1, *^, Weiqiang Chen^2, *^

^1^ Department of Microbiology and Immunology & Key Immunopathology Laboratory of Guangdong Province, Shantou University Medical College, Shantou, Guangdong, China.

^2^ Department of Neurosurgery, First Affiliated Hospital of Shantou University Medical College, Shantou, Guangdong, China.

^3^ Department of Neurosurgery, Second Affiliated Hospital of Shantou University Medical College, Shantou, Guangdong, China.

^4^ Department of Neurosurgery, Affiliated Jieyang Hospital of Sun Yat-sen University, Jieyang, Guangdong, China.

***Correspondence to**

**Weiqiang Chen,** MD &PhD, Department of Neurosurgery, First Affiliated Hospital, Shantou University Medical College, 57 Changping Road, Shantou, Guangdong, China. Email: [wqchen@stu.edu.cn](mailto:wqchen@stu.edu.cn); Tel: +86-0754-88905326.

**Kangsheng Li**, MD &PhD, Department of Microbiology and Immunology & Key Immunopathology Laboratory of Guangdong Province, Shantou University Medical College, 22Xinling Road, Shantou, Guangdong, China. Email: [ksli2013@yeah.net](mailto:ksli2013@yeah.net); Tel: +86-0754-88900840.

**Supplementary Table 1.** Baseline characteristics of patients with or without multihematoma fuzzy sign before and after propensity score (PS) matching in the validation cohort.

|  | **Before PS match** | | | **After PS match** | | |  |
| --- | --- | --- | --- | --- | --- | --- | --- |
| **Variables** | **No multihematoma fuzzy sign (n =89)** | **Multihematoma fuzzy sign (n = 75)** | ***P*-value** | **No multihematoma fuzzy sign (n = 50)** | **Multihematoma fuzzy sign (n =50)** | ***P*-value** |  |
| Male sex, no. (%) | | 65 (73.03%) | 56 (74.67%) | 0.813 | 37 (74.00%) | 35 (70.00%) | 0.656 |
| Mean age (SD), y | | 50.64 (17.11) | 51.91 (17.97) | 0.645 | 50.14 (17.46) | 50.32 (17.35) | 0.959 |
| Hypertension, no. (%) | | 12 (13.95%) | 14 (19.44%) | 0.354 | 8 (16.00%) | 8 (16.00%) | 1.000 |
| Diabetes, no. (%) | | 2 ( 2.30%) | 5 ( 6.85%) | 0.161 | 2 ( 4.00%) | 3 ( 6.00%) | 0.646 |
| Mean arterial pressure, median (IQR), mmHg | | 99.67 (88.67-109.33) | 100.00 (89.33-115.50) | 0.075 | 100.00 (90.75-110.58) | 98.84 (89.00-104.17) | 0.670 |
| Coagulopathy, no. (%) | | 11 (13.10%) | 11 (15.28%) | 0.696 | 8 (16.00%) | 8 (16.00%) | 1.000 |
| Level on Glasgow Coma Scale score, no. (%) | |  |  | 0.548 |  |  | 0.396 |
| Mild (13–15 points) | | 48 (53.93%) | 34 (45.33%) |  | 28 (56.00%) | 26 (52.00%) |  |
| Moderate (9–12 points) | | 16 (17.98%) | 16 (21.33%) |  | 6 (12.00%) | 11 (22.00%) |  |
| Severe (3–8 points) | | 25 (28.09%) | 25 (33.33%) |  | 16 (32.00%) | 13 (26.00%) |  |
| Location, no. (%) | |  |  | 0.035 |  |  | 0.974 |
| Frontal | | 35 (39.33%) | 42 (56.00%) |  | 26 (52.00%) | 25 (50.00%) |  |
| Temporal | | 40 (44.94%) | 31 (41.33%) |  | 19 (38.00%) | 23 (46.00%) |  |
| Parietal | | 3 ( 3.37%) | 1 ( 1.33%) |  | 1 ( 2.00%) | 1 ( 2.00%) |  |
| Occipital | | 5 ( 5.62%) | 1 ( 1.33%) |  | 2 ( 4.00%) | 1 ( 2.00%) |  |
| Basal ganglia, brainstem, or cerebellum | | 6 ( 6.74%) | 0 ( 0.00%) |  | 2 ( 4.00%) | 0 ( 0.00%) |  |
| Intraventricular hemorrhage, no. (%) | | 5 ( 5.62%) | 5 ( 6.67%) | 0.780 | 3 ( 6.00%) | 2 ( 4.00%) | 0.646 |
| Subarachnoid hemorrhage, no. (%) | | 67 (75.28%) | 63 (84.00%) | 0.170 | 38 (76.00%) | 39 (78.00%) | 0.812 |
| Subdural hemorrhage, no. (%) | | 61 (68.54%) | 64 (85.33%) | 0.012 | 40 (80.00%) | 40 (80.00%) | 1.000 |
| Time to baseline CT (IQR), h | | 3.00 (1.67-4.50) | 3.00 (1.92-5.00) | 0.679 | 2.88 (1.71-4.75) | 2.66 (1.83-4.63) | 0.659 |
| Time from baseline CT to follow-up CT (IQR), h | | 17.75 (10.38-24.00) | 14.25 ( 6.62-24.00) | 0.875 | 16.50 (9.87-23.75) | 10.68 (6.03-24.00) | 0.546 |
| Baseline tICH volume, mean (SD), mL | | 8.48 (9.71) | 11.46 (9.55) | <0.001 | 10.61 (12.00) | 10.33 ( 7.83) | 0.890 |
